# Does the choice of syndesmotic screw versus suture button in ankle surgery has a silver lining? – a technical note

**DOI:** 10.1186/s40634-020-00279-x

**Published:** 2020-09-13

**Authors:** Pieter D’Hooghe, Motasem Salameh

**Affiliations:** 1 Department of Orthopaedic Surgery, Aspetar Orthopaedic and Sports Medical Hospital, Sports City Street 1, P.O. Box 29222, Doha, Qatar; 2 Department of Orthopaedic Surgery, Hamad General Hospital, Doha, Qatar

**Keywords:** Ankle fracture, Athlete, Football, Return to play, Screw, Suture button, Syndesmosis

## Abstract

**Purpose:**

Unstable ankle fractures with associated syndesmotic injury are of considerable morbidity in the professional athlete population. The use of dynamic suture button versus static syndesmotic screws fixation, rehabilitation protocols and timing to return to play are still areas of debate. We report the use of novel algorithm of sequential static and dynamic syndesmotic fixation in an elite football player with Weber C ankle fracture.

**Methods:**

The patient underwent open reduction and internal fixation for a weber C ankle fracture with associated syndesmotic and deltoid ligament injury. The osteosynthesis included lateral malleolus neutralizing plate, two syndesmotic screws and deltoid anchor repair. At 6 weeks post-operative both syndesmotic screws were removed and one suture button was implanted in the proximal screw hole. After the second operation the patient was allowed full weight bearing and range of motion in all direction with accelerated rehabilitation protocol.

**Results:**

The technique provided satisfactory results. At 4 month the player participated in a 90 min official football match. The fracture healed uneventfully with no recurrent syndesmotic diastasis.

**Conclusion:**

The presented technique of sequential dynamic and static fixation of associated syndesmotic injuries combined advantages of both syndesmotic screws and suture button implants. In an aim to allow earlier return to play in an elite football player. This opens the way for higher level of evidence clinical trials.

## Background

Ankle injuries are one of the most common sports injuries [9], of which unstable ankle fractures are considered a significant source of morbidity to our athlete population. Competitive athletes with associated syndesmotic injury were found less likely to return to play at 1 year [2]. Although an evidence-based guideline for the management of acute ankle fractures in athletes is lacking, open reduction and internal fixation with 1 or 2 syndesmotic screws are the current golden standard treatment [14].

Ligamentous injuries are known to require more healing time compared to bony fractures. Hence, ankle fractures with combined syndesmotic injury require a prolonged period of rehabilitation [10]. As an early and safe return to play are the major focus when dealing with syndesmotic injuries in professional athletes, the use of dynamic vs rigid syndesmotic fixation is still debated. While screw fixation is proven to be more rigid [12], dynamic fixation with suture buttons allows for an earlier ankle mobility and weight bearing while maintaining the required reduction [11]. Furthermore, the need for early screw removal prior to weight bearing postoperatively, can lead to loss of reduction and remaining tibio-fibular diastasis [13].

Therefore, the authors present a novel fixation technique that uses a sequential rigid and dynamic syndesmotic fixation algorithm in an elite football player that sustained an acute ankle fracture with associated syndesmotic injury.

### Case and surgical technique

**Fig. 1:**
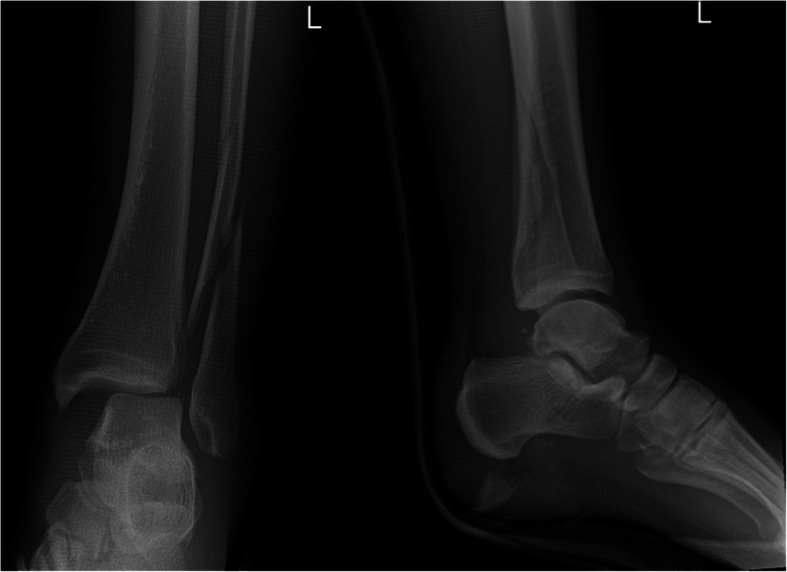
Anteroposterior and lateral plain ankle radiographs of the initial injury showing a Weber C ankle fracture subluxation with widened medial clear space, increased tibiofibular clear space, decreased tibiofibular overlap and lateral subluxation of the talus

A 27-year-old national team professional football player was referred to our institute after sustaining a left pronation external rotation ankle injury through a football tackle from behind. Figure 1 displays plain anteroposterior and lateral radiographs of the initial injury. This resulted in a Weber C ankle fracture subluxation requiring osteosynthesis. The radiographic parameters (widened medial clear space, increased tibiofibular clear space, decreased tibiofibular overlap and lateral subluxation of the talus) suggested an associated syndesmotic and deltoid ligament disruption injury pattern.

**Fig. 2:**
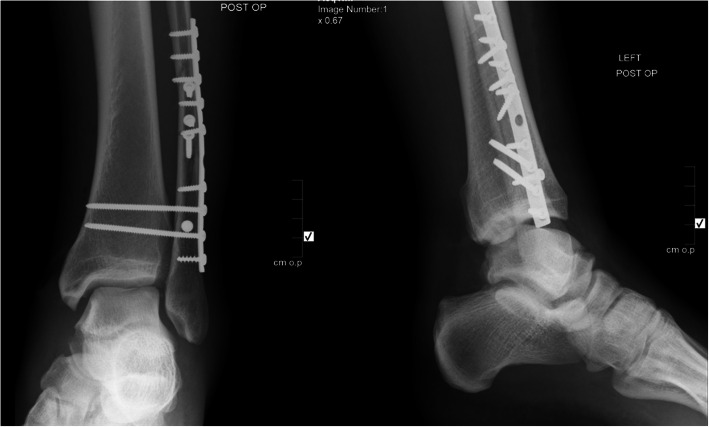
Post-operative anteroposterior and lateral plain ankle radiographs. Osteothynseis with lateral malleolus plate and two syndesmotic quadritcortical screws

The patient was positioned supine with a rolled towel under his left buttock to decrease the external rotation of the left lower limb. Access to the fracture via the direct approach to the lateral malleolus. The fibular length was restored and the fracture reduced and held with pointed bone reduction clamps. Fracture was fixed with four 3.5 mm cortical lag by technique screws and protected with 10-hole lateral neutralizing plate. After fixing the lateral malleolus, the external rotation and the hook tests were both positive. The syndesmosis was reduced under image intensifier control using a pelvic pointed clamp and fixed with two 3.5 mm quadricortical lag screws Fig. 2. A combined soft tissue anchor deltoid repair was added to the construct.

Post-operatively, the player was kept in a below knee cast with non-weight bearing physical therapy protocol for 14 days. Two weeks post-operatively, the cast was removed and partial weight bearing in a walking boot was initiated for the next 4 weeks. During these 4 weeks, the rehabilitation plan included sagittal range of motion, hydro gait therapy and anti-gravity treadmill training at 30% bodyweight.

**Fig. 3:**
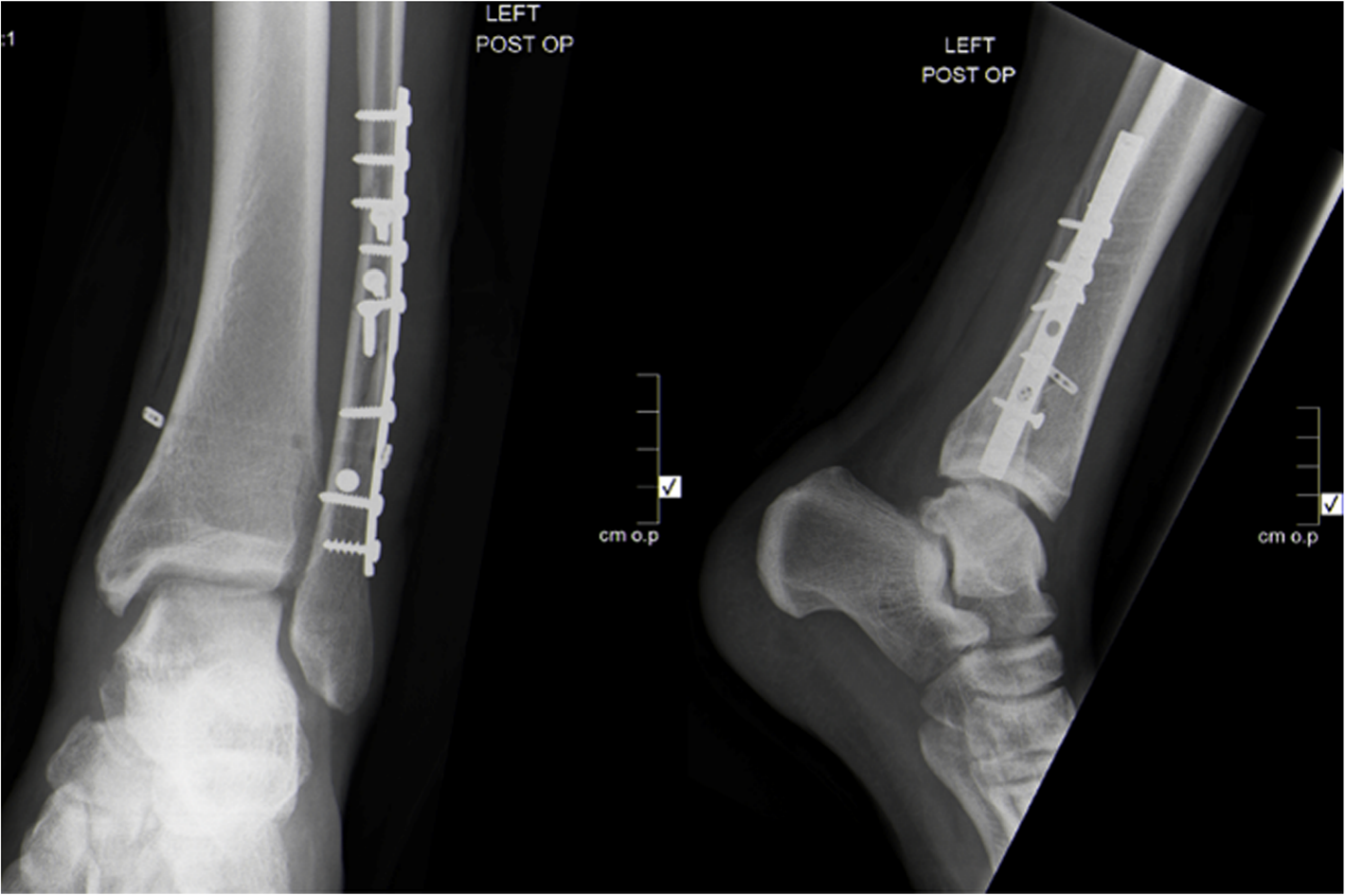
Anteroposterior and lateral plain ankle radiographs after the second operation demonstrating the exchange of the proximal syndesmotic screw with a suture button

At week 6 post-operatively, the patient was taken to the operating room for the removal of the two syndesmotic screws. A fluoroscopy-guided minimally invasive approach through part of the previous surgical incision was used. The upper syndesmotic screw was removed first and a suture button (Tightrope, Arthrex®) was inserted in the same hole, then the lower syndesmotic screw was removed and a short 3.5 mm screw was inserted. Figure 3 demonstrate postoperative radiographs with maintained syndesmotic reduction and no recurrent tibio-fibular diastasis.

Following review by the rehabilitation team after this second procedure, the patient was discharged day 1 post-operatively. Full weight bearing and full range of motion in all directions were started immediately and the patient was following daily rehabilitation in the outpatient physical therapy department. At 9 weeks after the initial injury, in collaboration with the team’s physician, the patient started single pitch training sessions and at 13 weeks he participated in his first group training session. At 4 months after the initial injury the patient successfully returned to play in a regular 90 min professional match. Since the player’s RTP online statistics showed that he has been regularly playing and scoring.

## Discussion

In this technical note, we present the case of a professional football player who sustained an unstable ankle fracture with associated syndesmotic injury. The proposed technique algorithm is based on a sequential rigid and dynamic syndesmotic fixation aiming for a faster rehabilitation and earlier return to play. While syndesmotic screws have long been the golden standard for fixation of syndesmotic injuries, suture buttons were introduced as a more physiological fixation method with a number of theoretical advantages [9].

Dynamic syndesmotic fixation has been suggested to allow for earlier postoperative range of motion and weight bearing while maintaining anatomical reduction with no concerns for implant failure and tibio-fibular diastasis [7]. Furthermore, there is a less risk of a symptomatic implant and need of second operation for removal. However, in a cadaveric study comparing different implants for syndesmotic fixation, syndesmotic screws were reported to offer the best rigid fixation with significantly less diastasis as compared to suture button fixation [12]. Teramoto et al. performed this study on 6 ankles comparing single suture button fixation, double suture button fixation, anatomic suture button and screw fixation. The authors evaluated the amount of diastasis with various stresses on the ankle. With single suture button the diastasis increased significantly with all forces. They also reported that the screw fixation proved to be the most rigid fixation, with significantly decreased diastasis. Another cadaveric study by Clanton et al. [3] reported the syndesmotic screw fixation provided the most rigid restraint to anterior- posterior fibular translation compared to single and double suture button, with the highest amount of translation seen in the single suture button construct.

A recent level 1 meta-analysis on 7 randomized controlled trials reported a lower complications rate and improved clinical outcomes in dynamic syndesmotic fixation as compared with static screw fixation. However, after limiting the analysis to only clinically relevant complications, no significant difference was found between the 2 implants. Furthermore, the superiority of suture buttons was found significant only when the analysis was based on studies with no routine screw removal [6]. In line with above, neither rigid nor dynamic fixation alone can combine the aim of rigid stable fixation and early weight bearing and ankle range of motion. Supporting our argument that combining both techniques in a sequential might be the silver lining for early rehabilitation and return to play in this type of injuries.

**Fig. 4:**
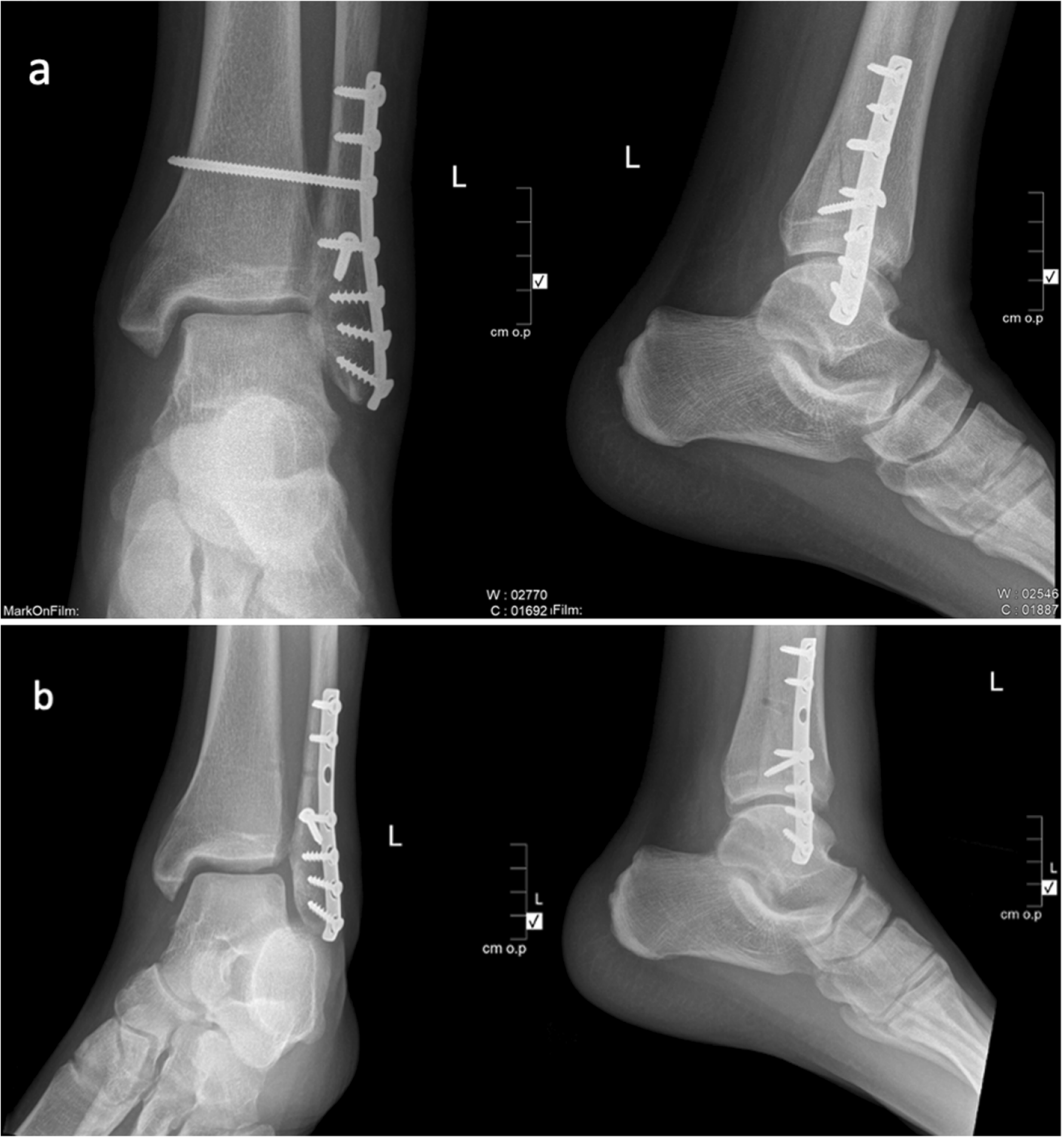
Example of recurrent diastasis after removal of syndesmotic screws. **a** Post-operative anteroposterior and lateral plain ankle radiographs demonstrating lateral malleolus neutralizing plate and syndesmotic screw fixation. **b** Post-operative anteroposterior and lateral plain ankle radiographs showing recurrent tibio-fibular diastasis after removal of the syndesmotic screw

The need for early weight bearing and rehabilitation in professional athletes mandates the routine early removal of syndesmotic screws in this particular high demand population. However, the risk of recurrent diastasis or remaining instability should be considered Fig. 4 . Moore et al. [11] and Hsu et al. [7] reported a higher incidence of recurrent syndesmotic diastasis when screws were removed between 6 and 8 weeks. Recently, Anderson et al. [1] reported a significant increased rate of syndesmotic diastasis at 1 year follow up after routine removal of syndesmotic screws at 10–12 weeks. This can be explained by the fact that the healing capacity of the ankle syndesmosis is significantly slower, requiring prolonged periods of fixation and non-weight bearing rehabilitation resulting in a delayed return to play.

The best timing for return to sports after unstable ankle fractures is still undefined, with data showing that professional athletes and those with syndesmotic injury were significantly less likely to return to sports [2]. Jelinak and Porter [8] suggested that successful return to play can take up to 6 months after syndesmotic screw fixation of unstable ankle fractures in professional athletes. In a recent study on return to play for surgically treated isolated unstable syndesmotic injuries, D’Hooghe et al. [4, 5] reported an early return to play with the first official match played on an average of 103 days.

A limitation of the current study is that it provides data of only one subject with a relatively short follow up. However, future higher level of evidence studies are needed to further validate our results.

## Conclusion

The presented technical note aims at sequentially combining the advantages of rigid and dynamic syndesmotic fixation to allow early return to play in athletes with unstable ankle fractures with syndesmotic injury. This needs further investigation by a higher level prospective large-scale trials.

## Data Availability

Data sharing is not applicable to this article as no datasets were generated or analyzed during the current study.
